# Upper and Lower Endoscopic Findings in Mesenteric Panniculitis Patients: A Case-Control Study

**DOI:** 10.3390/jcm13226709

**Published:** 2024-11-08

**Authors:** Hagai Schweistein, Yoav Weintraub, Tzipi Hornik-Lurie, Hassan Haskiya, Adi Rave, Ahinoam Glusman Bendersky, Nidal Issa, Timna Naftali, Rachel Gingold-Belfer

**Affiliations:** 1 Gastroenterology Division Rabin Medical Center—Beilinson Hospital, Petach Tikva 4941492, Israel; 2 School of Medicine, Faculty of Medical and Health Sciences, Tel Aviv University, Tel Aviv 6997801, Israel; 3 Research Authority, Meir Medical Center, Kfar Saba 4428164, Israel; 4 Department of Diagnostic Imaging, Meir Medical Center, Kfar Saba 4428164, Israel; 5 Department of Surgery Rabin Medical Center—Hasharon Hospital, Petach Tikva 49372, Israel; 6 Division of Gastroenterology, Meir Medical Center, Kfar Saba 4428164, Israel

**Keywords:** mesenteric panniculitis, colonoscopy, colon cancer, mortality

## Abstract

**Background**: The natural history and prognosis of mesenteric panniculitis (MP) are not well-described. Despite referral for colonoscopy being common for this indication, colonoscopy findings in MP patients have not been reported. Therefore, we aimed to describe upper and lower gastrointestinal (GI) endoscopy findings in patients with mesenteric panniculitis, compared to matched controls, to investigate their clinical outcomes including incidence of malignancy and mortality. **Methods**: Retrospective case–control study was conducted, and included patients who were diagnosed with mesenteric panniculitis according to Coulier radiologic criteria on abdominal computerized tomography between 1/2005 and 12/2019, and followed to 12/2021. The case group was compared to a matched control group without MP on abdominal CT. Clinical data and the upper and lower endoscopies’ reports were reviewed in both groups. We excluded patients who, beyond diagnosis of MP, were also diagnosed with current malignancy, significant intra-abdominal morbidity or inflammatory bowel disease. **Results**: The initial set of 376 patients with MP, after exclusion, included 187 patients. A total of 56.1% were male, with a mean age 60 ± 15 years. Of them, 74 (39%) patients underwent follow-up CT scans, which demonstrated, in 66 (89.2%) patients, a stable MP without any aggravation. Colonoscopy was performed in 89 MP patients, and 98/187 controls. No significant difference in the colonoscopies’ findings was found between the two groups. Gastroscopy was performed in 84 MP and 79 controls. No case of gastric cancer was found. No statistically significant difference was found in the rate of gastroscopy findings. By the end of the follow-up period, malignancy was diagnosed in four patients of the MP group. None were colon cancer. The mortality rate in the MP group was 3.2%, without a significant difference compared to the controls. None were MP related. **Conclusions**: MP identified on abdominal CT is not associated with pathologic endoscopy findings or future diagnosis of colon cancer, and also has no impact on mortality rate. Since repeating abdominal CT did not reveal any disease progression, the necessity of follow-up imaging for MP should be carefully reconsidered.

## 1. Introduction

Mesenteric panniculitis (MP) is characterized by changed levels of idiopathic fat necrosis, chronic inflammation, and fibrosis of the mesentery, primarily involving the small intestine [1,2]. The nomenclature surrounding MP has evolved to reflect the spectrum of disease severity and characteristics. Traditionally, MP described chronic, non-specific inflammation of the mesentery. Terms like sclerosing mesenteritis and retractile mesenteritis were used for more virulent presentations with increased fibrosis [1,2,3]. However, since 2019, Danford et al. suggested the use of “sclerosing mesenteritis” as a blanket term to describe the entire spectrum of benign fibro-inflammatory diseases of the mesentery [4,5]. This nomenclature is gaining momentum, but there is still significant variability between various sources of information.

Biopsy of the involved mesentery is considered the gold standard for diagnosing MP. However, the invasive nature of biopsy procedures has limited their use in confirming these diagnoses. Biopsy-based case series are rare and tend to be more progressive [5,6]. Consequently, imaging studies are the mainstay for diagnosis, and since 2011, radiologic criteria for MP identification, suggested by Coulier et al., are also widely used for diagnosis [4,7].

MP is considered an uncommon disease. Its prevalence ranges from 0.16%, when extracting the diagnosis by keywords from radiological assessments, to 2.5% in studies that assessed CT scans retrospectively [8,9,10,11,12].

However, nowadays, with the more widespread use of imaging [10], the prevalence of MP is increasing, and during recent years, a growing interest in this clinical entity has been emerging. In previous observational studies, the natural history of MP identified in imaging studies was usually benign, and follow-up CT scans did not demonstrate MP progression [4,8,10,11,13]. A few years ago, our group, investigated this entity too [12]. We conducted a matched case–control study which examined the relationship between MP and past medical histories and demonstrated that patients with MP had more cardiovascular risk factors compared to the general population and to a case–control group.

Our findings suggested that MP might have clinical significance and even might reflect a metabolic morbidity burden [12]. Similarly to other studies, we did not find a relationship between MP and a history of malignancy, surgeries or other morbidities [3,8,11,13,14].

No information exists on the results of upper and lower GI endoscopic examinations in this group of patients. There is evidence in the literature that links metabolic syndrome and obesity in particular, to the prevalence of adenomas in the colon [15,16] and also as a risk factor for cardiac gastric cancer [17]. Considering the findings in our previous study that metabolic syndrome and obesity are associated with MP, the question arose whether there is a higher prevalence of GI endoscopic findings, specifically colonic adenomas, colon cancer and gastric malignancy, in patients with MP compared to patients without MP.

Thus, we aimed to examine whether MP patients have more pathological findings on upper and lower GI endoscopies, by retrospectively comparing endoscopic results of MP patients with those of a matched control group.

Our secondary aims were to evaluate the outcomes and prognoses of patients with MP, including the incidence of malignancies and their mortality rate, compared to the control group. In addition, we investigated the progression of MP by evaluating the existence of changes in follow-up CT scans.

## 2. Patients and Methods

Meir Medical Center Ethics Committee approved this study. All patient data were anonymized. Given the registry-based design and anonymization of the data, informed consent was waived by the committee.

The study consisted of two distinct patient groups.

1. MP group: The database of Meir Medical Center was searched in a retrospective study, including all patients who underwent abdominal CT scans during hospitalization or emergency room visits from 1 January 2014 to 31 December 2019. Patients were identified if their abdominal CT report mentioned the following keywords: “panniculitis”, “MP” or “sclerosing mesenteritis” (in English or Hebrew). A specialist in abdominal CT reviewed the abdominal CT scans to confirm MP diagnosis based on the Coulier criteria [7], meaning 3 out of 5 criteria were fulfilled. Coulier et al.’s abdominal CT criteria are shown in Table 1. After the revision, we included only MP patients who met the Coulier criteria, and all the previous abdominal CT scans of the patient were retrospectively reviewed to determine the earliest time point at which MP was identified. The subsequent CT examinations of each patient were evaluated to check whether there was MP progression. Since we used the Coulier radiologic criteria to define our study group, we used the radiologic term “mesenteric panniculitis” to describe the disorder. Exclusion Criteria were as follows: patients with a documented active malignancy, inflammatory bowel disease (IBD), current dialysis, transplant history, abdominal surgery within the past six months, ischemic colitis, or intra-abdominal organ perforation. Patients with incomplete EMR data were also excluded. Study participants were followed until 31 December 2021.

2. Control group: A control group was established by matching each MP case with a control subject from the same database. Matching criteria were age, sex and year of abdominal CT examination. This process was performed by an external team of data monitors blinded to the investigators’ identities and MP diagnoses. To ensure a representative control group, benign CT findings such as cysts, hemangiomas, diverticula, and non-occlusive stones were not considered exclusionary. The same exclusion criteria applied to the MP group (active malignancy, IBD, dialysis, transplant, recent abdominal surgery, ischemic colitis, perforation) were also employed for the control group. The number of the matched controls was determined to be equal to the number of the cohort group to ensure the feasibility of the study, due to the limited size of Meir medical center database. In addition, the statistically most efficient approach is equal numbers of cases and controls, as had been published in previous studies [18,19].

## 3. Variables of Interest

Demographic and clinical data were extracted from the electronic medical records of Clalit Healthcare System (CHS), encompassing patients’ long-term medical history. Variables of interest included:

- Demographics: Age and sex.
- Endoscopy Reports: Data from colonoscopy and gastroscopy with biopsy reports were included when available. If multiple procedures were documented, only the most recent procedure (adjacent to the CT scan) was considered.
- Malignancy: Development of malignancy following MP diagnosis.
- Mortality: Patient mortality data.
- CT scans: Data encompassed:
  ○ Index CT (the initial CT scan in which MP diagnosis was given)
  ○ Prior CT scans
  ○ Subsequent CT scans

## 4. Statistical Analysis

The variables of interest were compared among the two groups using Pearson Chi-squared test for the categorical variables and Mann–Whitney test for the continuous and ordinal variables. All statistical tests were 2-sided and *p* < 0.05 was considered significant. SPSS version 25 software was used for data analysis (IBM Corp. Armonk, NY, USA). Kaplan–Meier survival analysis was used to generate survival curves. The log-rank test was used to test for significance.

## 5. Results

### 5.1. The Study Groups

A total of 376 CT scan records were initially identified based on the specified search terms. However, 37 patients’ medical files were inaccessible and therefore excluded from further analysis. An additional 82 patients were excluded due to pre-existing conditions, including active malignancy, inflammatory bowel disease, dialysis, transplant status, recent abdominal surgery, ischemic colitis, or perforation of an intra-abdominal organ. Of the remaining 257 CT scans, 70 patients did not meet the Coulier criteria for mesenteric panniculitis (MP) and were consequently excluded. This resulted in a final MP group comprising 187 patients, of whom 105 were male (56.1%) with a mean age of 60 ± 15 years (range: 25–103 years).

These patients were matched to 187 hospital patients without MP. The indications for performing abdominal CT in both groups were: abdominal pain, as part of a weight loss investigation or fever of unknown origin, elevation of liver enzymes, suspicion of appendicitis/cholecystitis/nephrolithiasis and MP follow-up.

**Figure 1:**
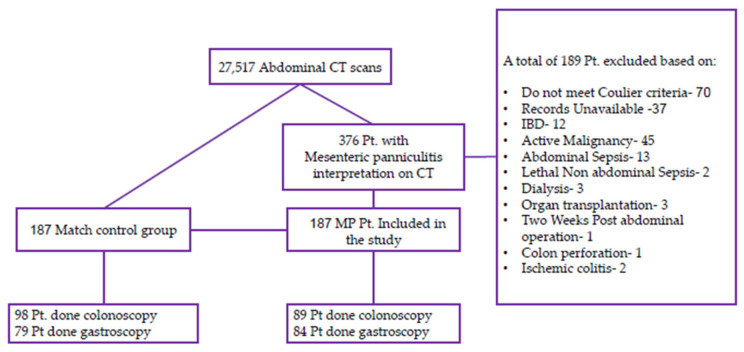
Flowchart of patients’ selection.

The flowchart for patient selection is shown in Figure 1.

### 5.2. Endoscopic Reports

#### Colonoscopy Findings

A total of 89 MP and 98 Non-MP patients had a colonoscopy report in the EMRs of CHS. When multiple colonoscopies were performed, data were extracted from the most recent colonoscopy proximate to the MP diagnosis. The median interval between index CT and adjacent colonoscopy was 13 months with an interquartile range of 4–34 months. No statistically significant difference was found in the colonoscopy results between the two groups. Table 2 shows the colonoscopy findings in each study group.

### 5.3. Gastroscopy Findings

A total of 84 MP and 79 Non-MP patients had a gastroscopy report in the EMR. No case of gastric cancer was found. Except for a higher report of gastric ulcers in the control group, without information on the prevalence of Helicobacter pylori in the two groups, no statistically significant difference was found in the rate of gastroscopy findings, as described in Table 3.

### 5.4. Malignancy and Mortality Rates

During a median follow-up of 4 years, with an interquartile range of 3–5 years, malignancy was diagnosed in four (2.1%) MP patients and in one (0.5%) control patient (*p* = 0.176). None were colon or gastric cancer.

**Figure 2:**
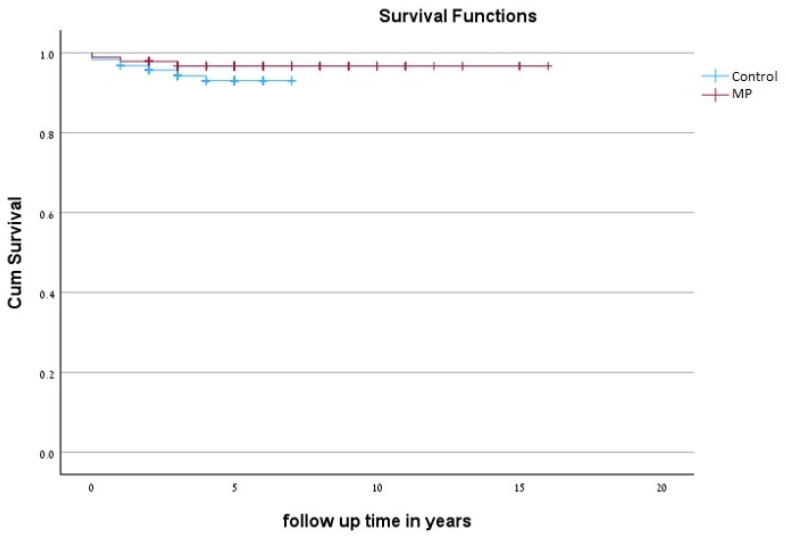
Kaplan–Meier survival curves for patients with Mesenteric Panniculitis compared to the matched control group.

By the end of the follow-up period, 6 and 11 patients of the MP and control groups, respectively, died. In the MP group four patients died because of sepsis, one as a result of cerebrovascular accident (CVA) and one for an unknown reason. In the control group, six patients died because of sepsis, one as a result of acute myocardial ischemia, one as a result of CVA, and three for unknown reasons. The mortality rate in the MP group was 3.2%, with no significant difference when compared to the control group, the mortality rate of which was 5.9% (*p* = 0.215) as shown in Table 4. None of the deaths were caused by complications commonly described in the literature as associated with mesenteric panniculitis, including bowel obstruction, bowel ischemia or perforation, and mortality during surgical intervention [6]. The mean age at death did not differ significantly between the MP group (84.5 ± 7.34 years) and the control group (77.09 ± 12.9 years), as indicated by the Mann–Whitney U test (U = 18.5, *p* = 0.14). Figure 2 depicts the Kaplan–Meier survival curves for patients with MP compared to the control group.

### 5.5. Follow-Up Abdominal CT Scans

By the end of the follow-up period, 74 MP patients (39%), underwent consecutive follow-up abdominal CT scans. A total of 33 patients had more than one CT. The mean interval between consecutive CT scans was 4.6 ± 3.3 years. Expert radiologist review demonstrated that the MP state was without progression in 66 (89.2%) of patients.

## 6. Discussion

Previous studies did not address the GI endoscopic findings, or the role of colonoscopy in the surveillance of MP patients. Thus, our study is the first study that investigated this issue. In this case–control study, we demonstrated that the diagnosis of idiopathic MP, according to the Coulier criteria, is not associated with adverse colonoscopy or gastroscopy findings, specifically colonic or gastric cancer, and no increased prevalence of colonic adenomas was observed. In addition, the gastroscopy reports did not reveal any association of MP with inflammation of the upper gastrointestinal track. These findings are in agreement with the existing research on the topic that does not link MP to malignancies in general and to colorectal malignancies in particular [4,11,14,20,21].

Together, our findings emphasize that from the clinical aspect, diagnosis of idiopathic MP by itself does not justify the performance of colonoscopies or gastroscopies. However, it is important to note that this conclusion should be applied with caution, as clinicians should initially classify idiopathic MP based on clinical background and imaging findings.

Our study also demonstrates that the mortality rate in MP patients is not increased. During a median follow-up of 4 years, with an interquartile range of 3–5 years, the mortality rate in the MP group was low and not related to the MP diagnosis, and the average age of death was 84.5 years; comparison of the mortality rate of MP patients with the control group demonstrated no difference. Our findings are also in agreement with the accepted mortality rate of the general population in Israel as reported by the Central Bureau of Statistics in their 2023 Statistical Abstract—https://www.cbs.gov.il/en/publications/Pages/2023/Statistical-Abstract-of-Israel-2023-No-74.aspx, accessed on 8 May 2024.

The practical clinical aspect of our study is the reassuring information that use of the Coulier criteria to establish the diagnosis of MP, in the appropriate clinical setting, may differentiate between benign MP and MP that requires more aggressive investigation. Prospective, double-blind studies are required to validate the predictive value of Coulier’s criteria for stratifying asymptomatic patients. These studies will enable us to distinguish between patients who do not necessitate further workups and those who require intervention and comprehensive evaluation. Furthermore, there is potential to develop a composite scoring system incorporating both clinical and imaging data, analogous to the model proposed by Danford et al. [4]. Beyond excluding secondary causes of MP, a history of metabolic syndrome should be included as a risk factor for idiopathic MP in the diagnostic algorithm. Additionally, future research should focus on enhancing the diagnostic accuracy of imaging to differentiate between benign and aggressive disease. Given its accessibility and safety, abdominal ultrasound presents a promising modality for future studies to investigate for its ability to discriminate between the two subtypes of mesenteric panniculitis [22,23].

The etiology of MP remains largely unknown. Given the increasing prevalence of abdominal imaging in contemporary healthcare settings, a promising avenue for future research would be to conduct large-scale cohort studies that prospectively evaluate individuals who have undergone abdominal imaging without evidence of MP and who then subsequently developed MP. By meticulously examining the medical records and imaging data of these individuals, we could potentially identify factors or events occurring between the two imaging studies that may have contributed to the onset and progression of the mesenteric inflammatory fibrotic processes. Such studies could help elucidate the temporal relationship between various clinical factors and the development of this condition, ultimately leading to a broader understanding of this phenomenon.

Our study has several limitations: The study was limited by the inherent biases of an observational retrospective design; however, the study participants of the two groups were followed past their index abdominal CT scans, by retrieving information from the CHS. The CHS database, continuously updated with real-time data from physicians and all health service providers, ensured the reliability and stability of the retrieved information.

Other limitations of the study are the relatively small number of patients from a single tertiary center and the limited follow-up period.

Despite these limitations, to our knowledge, this is the first original study addressing the role of GI endoscopic procedures in MP patients.

## 7. Conclusions

Our study reinforces the assumption that diagnosis of idiopathic MP is not related to malignancy in general or to colorectal cancer specifically. It is also not associated with increased or premature mortality. Thus, colonoscopy or gastroscopy are actually not indicated due to a diagnosis of idiopathic MP. Further, larger studies exploring the clinical significance of MP are warranted.

## Figures and Tables

**Table 1:** Coulier abdominal CT criteria for mesenteric panniculitis diagnosis *.

Number	Criteria
1	A mesenteric mass that clearly defines and displaces nearby structures without invading them
2	Inhomogeneous fat density within the mass greater than that of adjacent retroperitoneal/mesocolonic fat
3	The presence of small soft tissue nodules, typically less than 10 mm, within the mesenteric fat
4	A low-attenuation fatty halo surrounding the lymph nodes/mesenteric vessels
5	A hyperattenuating pseudo-capsule encircling the affected area in the absence of ascites or known malignancy involving the mesentery

* a total of 3 out of 5 criteria are needed to MP diagnosis establishment.

**Table 2:** Colonoscopy findings of patients with mesenteric panniculitis (MP) vs. control group.

Colonoscopy Findings	Patients with Computerized Tomography Results		*p*-Value
	MP (*n* = 89) N (%)	Control (*n* = 98) N (%)	
Tubular adenoma with low grade dysplasia below 1 cm	19 (21.3)	25 (26.0)	0.414
Malignancy/high-grade dysplasia	0	0	-
Diverticulosis	20 (22.5)	17 (17.7)	0.377
Non-adenomas polyps < 1 cm	7 (7.9)	15 (15.6)	0.121
Significant polyps (>1 cm)	3 (3.4)	2 (2.1)	0.565
Inflammatory findings in the terminal ileum	1 (1.1)	1 (1.0)	0.939

**Table 3:** Gastroscopy findings of patients with mesenteric panniculitis (MP) vs. control group.

Gastroscopy Findings	Patients with Computerized Tomography Results		*p*-Value
	MP (*n* = 84) N (%)	Control (*n* = 79) N (%)	
Gastric ulcer	2 (2.4)	9 (11.4)	0.023
Duodenal ulcer	2 (2.4)	5 (6.3)	0.22
Esophagitis	7 (8.3)	6 (7.6)	0.844
Duodenitis	13 (15.5)	9 (11.4)	0.428
Diaphragmatic hernia	18 (21.4)	22 (27.8)	0.273
Gastric polyp	3 (3.6)	8 (10.1)	0.100

**Table 4:** Rate of malignancy and mortality in patients with mesenteric panniculitis (MP) vs. control group during a median follow-up of 4 years, with an interquartile range of 3–5 years.

Variables	Patients with Computerized Tomography Results		*p*-Value
	MP (*n* = 187) N (%)	Control (*n* = 187) N (%)	
Colorectal cancer	0	0	-
Gastric cancer	0	0	-
Other malignancy	4 (2.1)	1 (0.5)	0.176
Mortality	6 (3.2)	11 (5.9)	0.215

## Data Availability

The data underlying this article cannot be shared publicly due to privacy requirements of participating centers. The data will be shared on reasonable request to the corresponding author.

## References

[1] Emory T.S., Monihan J.M., Carr N.J., Sobin L.H. (1997). Sclerosing mesenteritis, mesenteric panniculitis and mesenteric lipodystrophy: A single entity?. Am. J. Surg. Pathol..

[2] Eze V.N., Halligan S. (2023). Mesenteric panniculitis: A clinical conundrum. Br. J. Radiol..

[3] Green M.S., Chhabra R., Goyal H. (2018). Sclerosing mesenteritis: A comprehensive clinical review. Ann. Transl. Med..

[4] Danford C.J., Lin S.C., Wolf J.L. (2019). Sclerosing Mesenteritis. Am. J. Gastroenterol..

[5] Wagner C., Dachman A., Ehrenpreis E.D. (2022). Mesenteric panniculitis, sclerosing mesenteritis and mesenteric lipodystrophy: Descriptive review of a rare condition. Clin. Colon Rectal Surg..

[6] Akram S., Pardi D.S., Schaffner J.A., Smyrk T.C. (2007). Sclerosing mesenteritis: Clinical features, treatment, and outcome in ninety-two patients. Clin. Gastroenterol. Hepatol..

[7] Coulier B. (2011). Mesenteric panniculitis part 1: Mdct—Pictorial review. J. Belg. Soc. Radiol..

[8] Daskalogiannaki M., Voloudaki A., Prassopoulos P., Magkanas E., Stefanaki K., Apostolaki E., Gourtsoyiannis N. (2000). CT evaluation of mesenteric panniculitis: Prevalence and associated diseases. Am. J. Roentgenol..

[9] Wilkes A., Griffin N., Dixon L., Dobbs B., Frizelle F.A. (2012). Mesenteric panniculitis: A paraneoplastic phenomenon?. Dis. Colon Rectum..

[10] van Putte-Katier N., van Bommel E.F.H., Elgersma O.E., Hendriksz T.R. (2014). Mesenteric panniculitis: Prevalence, clinicoradiological presentation and 5-year follow-up. Br. J. Radiol..

[11] Gögebakan Ö., Albrecht T., Osterhoff M.A., Reimann A. (2013). Is mesenteric panniculitis truely a paraneoplastic phenomenon? A matched pair analysis. Eur. J. Radiol..

[12] Schweistein H., Weintraub Y., Hornik-Lurie T., Haskiya H., Levin S., Ringel Y., Naftali T. (2022). Mesenteric panniculitis is associated with cardiovascular risk-factors: A case-control study. Dig. Liver Dis..

[13] Coulier B. (2011). Mesenteric panniculitis. Part 2: Prevalence and natural course: MDCT prospective study. J. Belg. Soc. Radiol..

[14] Protin-Catteau L., Thiéfin G., Barbe C., Jolly D., Soyer P., Hoeffel C. (2016). Mesenteric panniculitis: Review of consecutive abdominal MDCT examinations with a matched-pair analysis. Acta Radiol..

[15] Wu H., Zhang J., Zhou B. (2021). Metabolic syndrome and colorectal adenoma risk: A systematic review and meta-analysis. Clin. Res. Hepatol. Gastroenterol..

[16] Shapero T.F., Chen G.I., Devlin T., Gibbs A., Murray I.C., Tran S., Weigensberg C. (2017). Obesity Increases Prevalence of Colonic Adenomas at Screening Colonoscopy: A Canadian Community-Based Study. Can. J. Gastroenterol. Hepatol..

[17] Thrift A.P., Wenker T.N., El-Serag H.B. (2023). Global burden of gastric cancer: Epidemiological trends, risk factors, screening and prevention. Nat. Rev. Clin. Oncol..

[18] Raju S.A., Greenaway E.A., Schiepatti A., Arpa G., Vecchione N., Jian C.L., Grobler C., Maregatti M., Green O., Bowker-Howel F.J. (2024). New entity of adult ultra-short coeliac disease: The first international cohort and case-control study. Gut.

[19] Verani J.R., Baqui A.H., Broome C.V., Cherian T., Cohen C., Farrar J.L., Feikin D.R., Groome M.J., Hajjeh R.A., Johnson H.L. (2017). Case-control vaccine effectiveness studies: Preparation, design, and enrollment of cases and controls. Vaccine.

[20] Buchwald P., Diesing L., Dixon L., Wakeman C., Eglinton T., Dobbs B., Frizelle F. (2016). Cohort study of mesenteric panniculitis and its relationship to malignancy. Br. J. Surg..

[21] Halligan S., Plumb A., Taylor S. (2016). Mesenteric panniculitis: Systematic review of cross-sectional imaging findings and risk of subsequent malignancy. Eur. Radiol..

[22] Whittle C., Schiappacasse G., Maldonado I., Villacres F., Hebel E., González F. (2022). Recognizing the ultrasound patterns of mesenteric panniculitis. Ultrasound Q..

[23] Biscaldi E., Romairone E., Rollandi G.A. (2002). Regarding six cases of mesenteric panniculitis: US, spiral CT, Magnetic Resonance. Radiol. Med..

